# Curative-Intention Surgery with Lobe-Specific Versus Systematic Lymph Node Dissection in Clinical Stage IA–IB Non-Small Cell Lung Cancer: Our Experience and Literature Review

**DOI:** 10.3390/healthcare13080957

**Published:** 2025-04-21

**Authors:** Bogdan Cosmin Tanase, Teodor Horvat, Mihnea Davidescu, Claudiu Eduard Nistor, Calin Muntean, Gabriel Veniamin Cozma, Alin Nicola, Felix Bratosin, Sorina Maria Denisa Laitin, Alin Burlacu

**Affiliations:** 1 Department of Thoracic Surgery, Prof. Alexandru Trestioreanu Institute of Oncology, 022328 Bucharest, Romania; bogdansen@gmail.com (B.C.T.); thorax_scumc@yahoo.com (T.H.); mihneadav@gmail.com (M.D.); alinburlacu@gmail.com (A.B.); 2 Department 4—Cardio-Thoracic Pathology, Thoracic Surgery II Discipline, Faculty of Medicine, “Carol Davila” University of Medicine and Pharmacy, 050474 Bucharest, Romania; ncd58@yahoo.com; 3 Medical Informatics and Biostatistics, Department III-Functional Sciences, “Victor Babes” University of Medicine and Pharmacy, 300041 Timisoara, Romania; 4 Thoracic Surgery Research Center, “Victor Babes” University of Medicine and Pharmacy, 300041 Timisoara, Romania; 5 Department of Surgical Semiology, “Victor Babes” University of Medicine and Pharmacy, 300041 Timisoara, Romania; 6 Doctoral School, “Victor Babes” University of Medicine and Pharmacy, 300041 Timisoara, Romania; alin.nicola@umft.ro; 7 Discipline of Thoracic Surgery, “Victor Babes” University of Medicine and Pharmacy, 300041 Timisoara, Romania; 8 Methodological Research Center for Infectious Diseases, “Victor Babes” University of Medicine and Pharmacy, 300041 Timisoara, Romania; felix.bratosin@umft.ro (F.B.); laitin.sorina@umft.ro (S.M.D.L.)

**Keywords:** lung cancer, oncology, thoracic surgery, mediastinal lymph nodes

## Abstract

Background and Objectives: The benefit of lobe-specific lymph node dissection (LS-LND) in non-small cell lung cancer (NSCLC) remains debated, especially in early-stage disease. Previous reviews often included all stages, leaving a gap in focused evaluations of clinical stage IA–IB NSCLC. This systematic review, supplemented by our institutional experience, aimed to compare overall survival (OS), recurrence-free survival (RFS), and postoperative complications between LS-LND and systematic lymph node dissection (S-LND) in clinical stage IA–IB NSCLC. Methods: We retrospectively reviewed 24 patients treated at our institution (14 S-LND vs. 10 LS-LND). Data on patient demographics, operative details, OS, RFS, and postoperative complications were collected. Risk of bias was assessed using established methodological tools. A targeted literature search was conducted in PubMed, EMBASE, and Web of Science from inception to 14 April 2022. Only three articles (total *n* = 1101 patients) met inclusion criteria focusing on clinical stage IA–IB NSCLC who underwent curative-intent resection. Results: LS-LND demonstrated comparable or slightly improved 5-year OS (range: 69.7–96.7%) versus S-LND (64.9–92.0%), and similar or slightly higher RFS (66.0–95.6% in LS-LND vs. 60.8–88.8% in S-LND). In our cohort, the 5-year OS was 78.6% in S-LND vs. 80.0% in LS-LND, and the 5-year RFS was 71.4% vs. 70.0%, respectively. Postoperative complications such as arrhythmias were less frequent in LS-LND groups overall. Our data showed a low rate of pneumonia in S-LND compared to LS-LND (7.1% vs. 10.0%); however, arrhythmias accounted for 14.3% in S-LND vs. 10.0% in LS-LND). Conclusions: For clinical stage IA–IB NSCLC, LS-LND offers oncologic outcomes that are comparable to S-LND, with a potential for reduced postoperative complications. The findings from our institution align with these trends observed in the literature. While these results suggest potential advantages of lobe-specific approaches, definitive conclusions require further validation through larger, prospective randomized studies to confirm the clinical benefits of LS-LND in early-stage NSCLC.

## 1. Introduction

Lung cancer remains one of the most common malignancies worldwide, representing a leading cause of cancer-related mortality [1]. Early-stage NSCLC is now detected not only through national low-dose CT screening programmes but also via adjunct advances such as integrated PET/CT fusion imaging, high-resolution thin-slice chest CT, and minimally invasive nodal staging with endobronchial-ultrasound-guided transbronchial needle aspiration (EBUS-TBNA), all of which collectively raise the sensitivity of the initial diagnostic work-up to well above 90% and refine both T- and N-category assessment [1,2]. Patients who present with clinical stage IA–IB disease often undergo surgical resection as the standard of care, yet the optimal surgical strategy for lymph node management remains unsettled [3]. Systematic lymph node dissection (S-LND), which entails the thorough removal of all ipsilateral mediastinal, hilar, and interlobar nodes, is traditionally performed to ensure adequate staging and local control [4]. However, the extensive nature of S-LND can potentially increase postoperative complications such as chylothorax and arrhythmias [5]. These complications may adversely affect long-term survival by increasing morbidity and occasionally delaying or complicating subsequent therapies [6]. Investigating more tailored approaches to lymph node management is therefore of high clinical relevance for thoracic surgeons, oncologists, and patients alike.

Lobe-specific lymph node dissection (LS-LND) has emerged as an alternative that selectively removes those mediastinal and hilar lymph node stations most likely to be involved, depending on the tumour’s lobe of origin [7]. Early proponents of LS-LND have suggested that tumours in certain lobes follow predictable pathways of lymphatic spread [8]. Hence, removing only the anatomically relevant stations could reduce operative time, minimize surgical trauma, and potentially lower the risk of complications while still maintaining oncologic efficacy [9]. In early-stage disease—particularly clinical stage IA–IB—the likelihood of extensive nodal spread is smaller compared to advanced stages [10]. Therefore, a selective or lobe-specific approach may be especially suitable for these patients.

Previous data often included patients with a broad range of tumour stages (IA–IIIA or even beyond) and consequently reported mixed findings on LS-LND outcomes [11,12]. As tumour stage increases, the potential benefits and risks of lymph node dissection may shift. For instance, in more advanced tumours, the risk of occult mediastinal involvement is higher, thus potentially favouring more extensive dissection [13]. Conversely, lower-stage tumours may experience fewer nodal metastases, making a less extensive dissection adequate for complete resection and accurate staging [14]. By isolating studies that focus strictly on stage IA–IB NSCLC, clinicians can gain a clearer picture of whether LS-LND confers distinct advantages—such as less morbidity—without compromising oncologic endpoints like survival.

Under the umbrella of clinical stage IA–IB, tumours are typically smaller (T1 or T2a) and lack clinical evidence of nodal (N0) or distant (M0) spread, as per the TNM staging system [15]. Accurate staging, however, hinges on thorough pathologic assessment of resected tissue, including the lymph nodes [16]. If LS-LND is sufficiently rigorous for these early lesions, patients could avoid added operative trauma and its consequences on quality of life. Traditional arguments against selective node dissection include the risk of understaging if unsuspected nodal involvement lies outside the “lobe-specific” stations [17]. This concern persists even in early-stage disease, though evolving imaging modalities, advanced pathology techniques, and better patient selection may reduce that risk. Because the nuances of LS-LND vary somewhat between institutions—particularly regarding which specific stations must be removed—there is also variability in how “lobe-specific” is defined [17,18].

We hypothesized that, in patients with clinical stage IA–IB NSCLC, lobe-specific lymph node dissection would provide oncologic outcomes—namely overall survival (OS) and recurrence-free survival (RFS)—that are at least equivalent to those achieved by systematic lymph node dissection, while reducing perioperative morbidity. Therefore, the primary study objective was to compare LS-LND and S-LND in early-stage NSCLC, focusing on 5-year OS, 5-year RFS, and postoperative complications. Through combining a comprehensive literature review with our own institutional data, this study aims to fill a gap in previous research, which often grouped patients across multiple stages; by limiting the analysis to clinical stage IA–IB disease, we bring a more precise evaluation of LS-LND’s potential advantages and its clinical feasibility in truly early-stage disease.

## 2. Materials and Methods

### 2.1. Study Design and Setting

This study was conducted at the Victor Babes University of Medicine and Pharmacy Timisoara, Romania. Ethical approval was granted by the institutional review board of Victor Babes University of Medicine and Pharmacy Timisoara. For the retrospective arm of this study, our institutional database was queried for patients who underwent curative-intent resection for clinically staged IA–IB NSCLC since 2018. Inclusion criteria required individuals to have a tumour classified as T1–T2a, N0, or M0 on preoperative imaging (computed tomography and PET-CT), and to have undergone either LS-LND or S-LND via standard lobectomy or anatomical segmentectomy. Patients were excluded if they had evidence of more advanced staging (beyond T2a or N0), multifocal disease, or significant comorbidities precluding surgery, or if they had incomplete medical records that prevented accurate assessment of postoperative outcomes. This approach ensured a well-defined, homogeneous cohort suitable for comparing the two lymph node dissection methods.

For the literature review, we searched the literature for studies with explicit inclusion of patients diagnosed with clinical stage IA–IB NSCLC undergoing LS-LND vs. S-LND. From the original databases (PubMed, EMBASE, and Web of Science), we identified which articles explicitly stated clinical stage IA–IB in their methods or patient selection criteria. Next, we screened article titles, abstracts, and full texts for relevant details on staging definitions. Only full-text, peer-reviewed publications reporting direct comparisons between LS-LND and S-LND and providing outcomes such as OS, RFS, or postoperative complications were retained. Conference abstracts, editorials, and review articles were excluded.

We extracted the study populations, surgical methods, pathological details, and results relevant to the narrower stage-based analysis. Where necessary, we contacted corresponding authors for clarification on staging definitions or numeric outcome data. Ultimately, this subset analysis reflects a narrow scope intended to inform surgeons on best practices for mediastinal lymph node management in early-stage NSCLC. Our methodology aligns with the PRISMA guidelines for systematic reviews [19], with adaptations to reflect the narrower inclusion criteria [20].

In keeping with the 8th edition of the AJCC/IASLC TNM classification for non-small cell lung cancer, stage I disease is confined to the lung parenchyma without nodal or distant spread (N0 M0) and is subdivided purely by primary-tumour (T) attributes. Stage IA encompasses tumours ≤ 3 cm in the greatest dimension: IA1 refers to T1a lesions ≤ 1 cm, IA2 to T1b lesions > 1 cm but ≤2 cm, and IA3 to T1c lesions > 2 cm but ≤3 cm. Stage IB corresponds to T2a tumours > 3 cm but ≤4 cm, or to tumours ≤ 4 cm that invade the visceral pleura, involve the main bronchus ≥ 2 cm distal to the carina, or are associated with segmental-level atelectasis/obstructive pneumonitis; by definition, these cases also remain N0 and M0. Thus, the clinical IA–IB cohort analysed here represents small (≤4 cm), node-negative, non-metastatic lesions in which therapeutic controversy centres on the optimal extent of lymph-node dissection rather than on systemic-therapy considerations (AJCC 8th ed., 2017).

Of 24 resections, three patients (2 pN1, 1 pN2) were pathologically up-staged and therefore excluded from the survival and complication analyses, leaving 21 true stage-I cases for comparison.

### 2.2. Data Extraction

**Figure 1:**
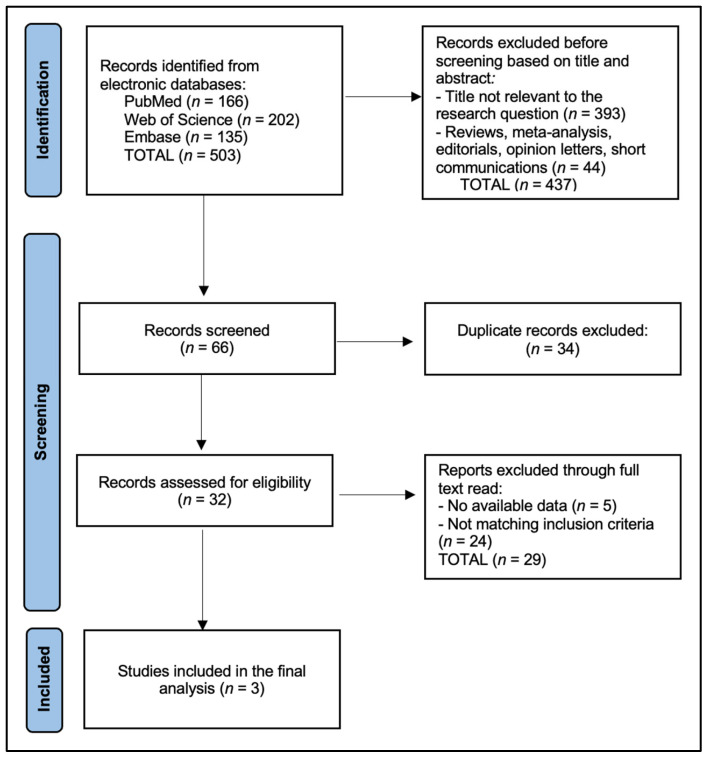
PRISMA flowchart.

Two reviewers independently performed data extraction from each of the included studies, based on the PRISMA flowchart presented in Figure 1. We developed a standardized extraction sheet that captured details on (1) baseline patient characteristics (age, sex, smoking status, comorbidities), (2) tumour characteristics (tumour diameter, histological subtype, radiological classification if applicable), (3) operative details (extent of resection, approach—open or minimally invasive, definition used for LS-LND, average lymph node stations sampled), and (4) key outcomes (5-year OS, RFS, postoperative complications, or any relevant numeric indicators of morbidity).

Whenever discrepancies arose in the data extraction process, a third reviewer was consulted to reconcile differences. For studies that presented survival curves rather than hazard ratios (HRs), we reported HRs and 95% confidence intervals (CIs). Complications were recorded in terms of frequency of pneumonia, chylothorax, arrhythmia, prolonged air leak, and early mortality. We coded all extracted variables in a single spreadsheet to ensure consistency across the final dataset.

### 2.3. Statistical Analysis

All statistical analyses were performed using IBM SPSS Statistics (version 27). Statistical significance was set at *p* < 0.05. Due to the limited number of studies and the heterogeneity in data reporting, we primarily performed a descriptive analysis. Any hazard ratios (HRs) and 95% confidence intervals (CIs) provided by the original studies were extracted. For complications, we summarized absolute numbers and percentages. We calculated 5-year OS and RFS by dividing the number of surviving or recurrence-free individuals by the total in each group (S-LND vs. LS-LND). No formal statistical pooling such as meta-analysis was performed.

## 3. Results

Table 1 shows four studies comparing S-LND and LS-LND in clinical stage IA–IB NSCLC. Hattori et al. (2021) [21] is a retrospective study from Japan (2008–2016) with 459 participants divided into 181 S-LND and 278 LS-LND. Zhao et al. (2021) [22] is a retrospective study from China (2014–2017) with 546 participants, where 446 underwent S-LND and 100 received LS-LND. Ma et al. (2013) [23] is a prospective study from China (2004–2008) with 96 participants (51 S-LND vs. 45 LS-LND). The current study is a retrospective study from Romania (2018–2020) involving 24 participants (14 S-LND vs. 10 LS-LND).

Table 2 presents baseline patient characteristics across the four studies. Hattori et al. (2021) [21] reported mean ages of 66 vs. 65 years for S-LND and LS-LND, with 64.1% vs. 67.6% males, and 70.7% vs. 68.7% adenocarcinomas. Zhao et al. (2021) [22] showed mean ages of 58 vs. 57, male percentages of 47.8% vs. 36.0%, and adenocarcinoma rates of 80.7% vs. 92.0%. Ma et al. (2013) [23] did not report male percentages or adenocarcinoma rates but indicated a mean age of 60 vs. 59 years. The current study documented mean ages of 57 vs. 60, male proportions of 50.0% vs. 40.0%, and adenocarcinoma percentages of 71.4% vs. 70.0% for S-LND vs. LS-LND.

**Figure 2:**
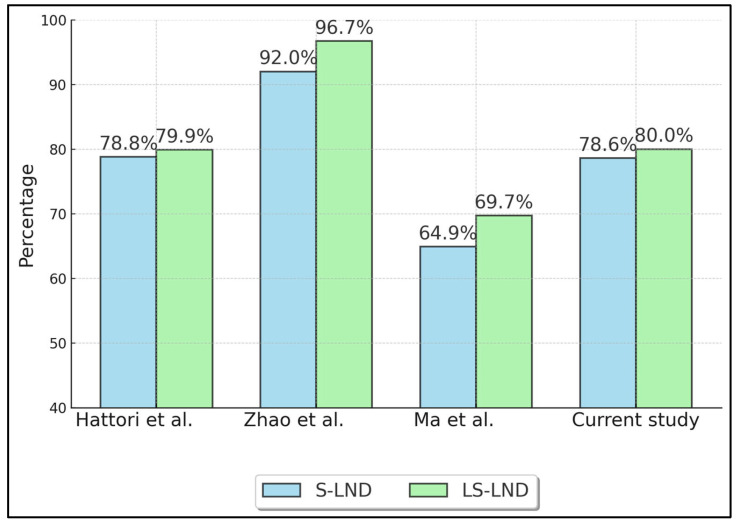
Five-Year Overall Survival (OS). Hattori et al. (2021) [21], Zhao et al. (2021) [22], Ma et al. (2013) [23].

**Figure 3:**
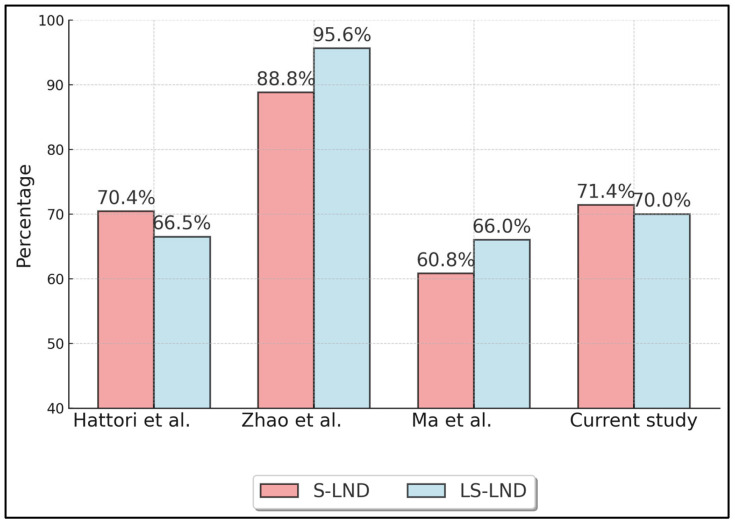
Five-Year Recurrence-Free Survival (RFS). Hattori et al. (2021) [21], Zhao et al. (2021) [22], Ma et al. (2013) [23].

Table 3 summarizes oncologic outcomes, specifically 5-year overall survival (OS) (Figure 2) and recurrence-free survival (RFS) (Figure 3) for S-LND versus LS-LND, along with *p*-values. Hattori et al. (2021) [21] reported 78.8% vs. 79.9% OS, and 70.4% vs. 66.5% RFS, with *p*-values of 0.665 and 0.669, respectively. Zhao et al. (2021) [22] showed 92.0% vs. 96.7% OS and 88.8% vs. 95.6% RFS, with *p*-values of 0.411 and 0.13. Ma et al. (2013) [23] indicated 64.9% vs. 69.7% OS, 60.8% vs. 66.0% RFS, and *p*-values of 0.552 and 0.241. The current study reported 78.6% vs. 80.0% OS and 71.4% vs. 70.0% RFS, with *p*-values of 0.892 and 0.911.

Table 4 focuses on postoperative complications across the four studies. Hattori et al. (2021) [21] noted pneumonia rates of 4.9% vs. 3.2%, arrhythmias of 13.3% vs. 10.1%, and early mortality of 1/181 vs. 3/278; chylothorax was not reported (NR). Zhao et al. (2021) [22] did not report pneumonia or chylothorax rates but showed arrhythmias of 3% vs. 1% and no data on early mortality. Ma et al. (2013) [23] reported pneumonia in 9.8% vs. 4.4%, arrhythmias in 4% vs. 2.2%, chylothorax in 4% vs. 0, and no early mortality data. The current study showed pneumonia in 7.1% vs. 10.0%, arrhythmias in 14.3% vs. 10.0%, no chylothorax in either group, and no early mortality.

## 4. Discussion

### 4.1. Analysis of Findings

These findings underscore that within clinical stage IA–IB NSCLC, LS-LND delivers comparable, if not slightly improved, oncologic outcomes relative to the more extensive S-LND. Across the three included studies, 5-year OS differences were mostly not statistically significant, although Zhao et al. [22] reported a numerical edge favouring LS-LND. In our series, median LOS was 7 days (IQR 6–9) after S-LND versus 5 days (IQR 4–7) after LS-LND (*p* = 0.28), mirroring IA–IB data from Hattori (7 vs. 6 days) and Zhao (6.4 ± 2.1 vs. 5.2 ± 1.9 days). This suggests that for small, localized tumours, the additional mediastinal clearance achieved with S-LND may not confer a clear survival advantage. From a pathophysiological standpoint, early-stage lesions have limited nodal spread, so a lobe-specific approach can adequately address the most relevant stations [24]. A parallel benefit hinted at is the trend toward fewer postoperative complications with LS-LND. Reduced manipulation of mediastinal structures likely underlies the slightly lower incidences of arrhythmias and chylothorax reported, translating into shorter hospitalizations and potentially lower costs.

Regarding complications, post-operative atrial fibrillation (POAF) reflects autonomic-nerve injury, atrial stretch, and inflammatory surge that accompany lung resection; systematic mediastinal lymph-node dissection magnifies these triggers because stripping tissue around the vagus, cardiac plexus, and pulmonary-vein sleeves adds operative trauma and cytokine release. A classic 267-patient analysis found arrhythmias in 23.6% of thoracic procedures and noted a clear excess when mediastinal nodes were removed [25], and more recent Japanese data likewise identified mediastinal dissection as an independent POAF predictor, prompting calls to omit it in high-risk stage I cases [26]. Mechanistic studies link direct vagal/pericardial stimulation during node clearance to ectopic firing near pulmonary veins, explaining why lobe-specific dissections that spare these fibres consistently show lower POAF rates [27]. In parallel, a 53,000-patient cohort demonstrated that elevated post-operative C-reactive protein independently predicts POAF, highlighting the inflammatory component that extensive nodal clearance exacerbates [28]; together, these data suggest that limiting dissection to lobe-specific stations can shorten operative time, blunt inflammation and roughly halve arrhythmia risk without sacrificing oncologic staging.

Nonetheless, important questions remain. “Lobe-specific” dissection was not identically defined across studies; Zhao et al. [22] required fully solid IA tumours, whereas Ma et al.’s [23] older dataset may not reflect contemporary imaging and pathology standards. Hattori et al. [21] had the largest cohort but still concluded that OS and RFS were broadly equivalent. Variations in staging technology, surgical expertise, and pathology processing therefore temper the generalisability of current evidence. Skip-metastasis to uninvolved mediastinal stations remains a theoretical concern, yet none of the analysed cohorts showed an excess of locoregional recurrence after LS-LND, supporting the oncologic safety of a selective strategy for truly early-stage disease. This concern is greatest for right-upper-lobe tumours (stations 2R/4R) and lower-lobe tumours of either lung (station 7), where skip N2 involvement is most frequently reported.

Large observational series beyond stage I bolster these observations. For hyper-metabolic T2–T3 tumours, Handa et al. found a numerical (though not statistically confirmed) CSS advantage for systematic LND, whereas Kuroda et al. and Adachi et al. detected no survival penalty with lobe-specific or selective dissection after propensity matching [24,29,30,31]. Together, these reports suggest that the therapeutic margin favouring systematic LND narrows as tumour burden decreases and imaging accuracy improves.

Similarly, it is important to mention that Mark Shapiro et al. [32] conducted a study analysing the efficacy of lobe-specific mediastinal nodal dissection in early-stage NSCLC, suggesting that lobe-specific evaluation during lobectomy could be adequate given the predictable patterns of nodal disease. From July 2004 to April 2011, they assessed 370 patients, with findings indicating that the recurrence rates for complete systematic lymph node sampling (20.6%) were comparable to those of lobe-specific evaluation (18.2%, *p* = 0.68). Furthermore, the occurrence of N2 disease was relatively low (5.3% in complete SLNS group), demonstrating that lobe-specific mediastinal evaluation could be sufficient for early-stage NSCLC patients with negative preoperative scans. In a similar manner, but applying a more rigorous methodological approach using propensity score matching, Futoshi Ishiguro et al. [33] evaluated the effects of selective versus complete mediastinal lymph node dissection in a larger cohort from 1995 to 2003. Their study involved 772 patients and showed no significant difference in 5-year overall survival between selective (76.0%) and complete dissection (71.9%). Both studies, thus, support the potential adequacy of less extensive nodal dissection in specific NSCLC populations, with Ishiguro et al.’s findings providing additional validation through advanced statistical adjustments, reflecting no significant detriment in survival outcomes with selective dissection [33]. This cumulative evidence may inform surgical decision-making, emphasizing tailored approaches based on individual nodal disease patterns and surgical risk-benefit considerations.

### 4.2. Implications for Guidelines and Clinical Practice

Current NCCN and ESTS/ERS guidelines still recommend systematic mediastinal node dissection (or at minimum systematic sampling) during anatomic lung resection for cN0 NSCLC, largely to ensure accurate staging and to guide adjuvant therapy decisions [34,35]. However, neither document differentiates recommendations by tumour size or lobe-specific drainage patterns. The evidence presented in this study suggests that, for carefully selected cIA–IB tumours ≤ 2 cm with negative PET/CT and EBUS findings, LS-LND may offer a non-inferior oncologic outcome while reducing operative time and peri-operative morbidity. This aligns with emerging data indicating that examining 8–11 nodes is sufficient for precise staging in stage I disease [36]. 

In practice, implementing a tiered strategy could mirror the evolution seen in breast cancer (sentinel node biopsy) and gastric cancer (D1+ vs. D2 dissection). Surgeons operating in high-volume centres with established quality metrics might adopt LS-LND as the default for peripheral tumours < 2 cm, escalating to systematic LND when intra-operative frozen-section evaluation shows unexpected nodal disease, when the tumour is central, or when pre-operative imaging raises suspicion for skip-metastasis. For low-volume institutions, adherence to systematic dissection may remain prudent until auditing confirms equivalent nodal harvest and recurrence rates.

Guideline panels may therefore consider risk-stratified recommendations that distinguish tumours ≤2 cm with peripheral location from larger or centrally situated lesions. Similarly, consideration should be given to minimum-node thresholds independent of dissection type (e.g., ≥8 nodes), focusing on pathologic adequacy rather than surgical extent. Reporting standards requiring explicit documentation of lobe-specific stations sampled should be considered to facilitate registry-based outcome tracking. Incorporating these nuances could harmonise surgical practice with contemporary imaging capabilities and enhance shared decision-making by offering patients an evidence-based, morbidity-sparing option without compromising oncologic integrity.

### 4.3. Study Limitations

First, only three stage-I-focused studies met inclusion criteria, and two were retrospective, leaving room for selection bias and residual confounding. Sample sizes in LS-LND arms remained modest, limiting statistical power. Radiologic and pathologic definitions varied, and important confounders (smoking status, comorbidity indices, ECOG performance) were incompletely reported. Complication definitions lacked uniformity, impeding pooled analyses. Finally, most contemporary guideline statements derive from mixed-stage populations; prospective randomised trials restricted to cIA–IB patients are still needed to validate the safety and long-term efficacy of LS-LND before universal adoption.

## 5. Conclusions

In clinical stage IA–IB NSCLC, lobe-specific lymph node dissection (LS-LND) provides oncologic outcomes that are comparable to those achieved with systematic lymph node dissection (S-LND). The analysis of three studies alongside our institutional data does not reveal significant differences in 5-year survival or recurrence-free survival (RFS); however, there is an indication of potentially fewer complications associated with LS-LND. The outcomes observed in our cohort of 24 patients further support these trends, demonstrating similar 5-year OS and RFS, with low morbidity rates noted in both LS-LND and S-LND groups. To firmly establish the role of LS-LND in clinical practice, further research involving larger, prospective randomized studies is essential to confirm its benefits and refine guidelines for optimal patient selection and surgical strategies in early-stage NSCLC.

## Figures and Tables

**Table 1:** Study Characteristics.

Study (Year)	Country	Design	Study Period	Number of Participants (S-LND vs. LS-LND)
Hattori et al. (2021) [21]	Japan	Retrospective	2008–2016	459 (181 vs. 278)
Zhao et al. (2021) [22]	China	Retrospective	2014–2017	546 (446 vs. 100)
Ma et al. (2013) [23]	China	Prospective	2004–2008	96 (51 vs. 45)
Current study	Romania	Retrospective	2018–2020	24 (14 vs. 10)

S-LND, Standard Lymph Node Dissection; LS-LND, Lobe-Specific Lymph Node Dissection.

**Table 2:** Baseline Patient Characteristics.

Study	Mean Age (Years) (S-LND vs. LS-LND)	Male (%) (S-LND vs. LS-LND)	Adenocarcinoma (%) (S-LND vs. LS-LND)
Hattori et al. (2021) [21]	66 vs. 65	64.1 vs. 67.6	70.7 vs. 68.7
Zhao et al. (2021) [22]	58 vs. 57	47.8 vs. 36.0	80.7 vs. 92.0
Ma et al. (2013) [23]	60 vs. 59	Not reported	Not reported
Current study	57 vs. 60	50.0 vs. 40.0	71.4 vs. 70.0

Not reported (NR) indicates data not available in the study. Abbreviations: S-LND, Standard Lymph Node Dissection; LS-LND, Lobe-Specific Lymph Node Dissection.

**Table 3:** Oncologic Outcomes: Five-Year Overall Survival (OS) and Recurrence-Free Survival (RFS).

Study	5-Year OS (S-LND vs. LS-LND)	5-Year RFS (S-LND vs. LS-LND)	*p*-Value (OS)	*p*-Value (RFS)
Hattori et al. [21]	78.8% vs. 79.9%	70.4% vs. 66.5%	0.665	0.669
Zhao et al. [22]	92.0% vs. 96.7%	88.8% vs. 95.6%	0.411	0.13
Ma et al. [23]	64.9% vs. 69.7%	60.8% vs. 66.0%	0.552	0.241
Current study	78.6% vs. 80.0%	71.4% vs. 70.0%	0.892	0.911

OS, Overall Survival; RFS, Recurrence-Free Survival; S-LND, Standard Lymph Node Dissection; LS-LND, Lobe-Specific Lymph Node Dissection.

**Table 4:** Postoperative Complications.

Study	Pneumonia (S-LND vs. LS-LND)	Arrhythmia (S-LND vs. LS-LND)	Chylothorax (S-LND vs. LS-LND)	Early Mortality (S-LND vs. LS-LND)
Hattori et al. [21]	4.9% vs. 3.2%	13.3% vs. 10.1%	NR	0.5% vs. 1.1%
Zhao et al. [22]	NR	3% vs. 1%	NR	NR
Ma et al. [23]	9.8% vs. 4.4%	4% vs. 2.2%	4% vs. 0%	NR
Current study	7.1% vs. 10.0%	14.3% vs. 10.0%	0% vs. 0%	0% vs. 0%

NR indicates ‘Not Reported’. Early Mortality is reported as the number of deaths per the total number of participants in each group. Abbreviations: S-LND, Standard Lymph Node Dissection; LS-LND, Lobe-Specific Lymph Node Dissection.

## Data Availability

The data presented in this study are available on request from the corresponding author.
